# Psychological Impact and Women’s Evaluation of the First-Trimester Pre-Eclampsia Screening and Prevention: ASPRE Trial

**DOI:** 10.3390/ijerph20075418

**Published:** 2023-04-06

**Authors:** Ana V. Nikčević, Chiara Sacchi, Claudia Marino, Neil O’Gorman, Liona C. Poon, Kypros H. Nicolaides

**Affiliations:** 1 Department of Psychology, Kingston University, London KT1 2EE, UK; 2 Department of Developmental and Social Psychology, University of Padova, 35121 Padova, Italy; 3 Coombe Women and Infants University Hospital, D08 XW7X Dublin, Ireland; 4 Department of Obstetrics and Gynaecology, The Chinese University of Hong Kong, Hong Kong, China; 5 Harris Birthright Research Centre of Fetal Medicine, King’s College Hospital, London SE5 8BB, UK

**Keywords:** psychological impact, pre-eclampsia, screening, ASPRE

## Abstract

Objective: This study aims to extend the understanding of the psychological impact of the first-trimester pre-eclampsia (PE) screening on women identified as high risk for preterm PE. We examined the differences between low- vs. high-risk women throughout pregnancy in: symptoms of distress (anxiety, depression, physical and mental health, and worry), health behaviour changes, the experience of pregnancy, and attitudes towards PE screening. Methods: This study was nested within the ASPRE trial. Pregnant women were screened for preterm-PE risk status in the first trimester; the assessments were carried out before the screening, in the second and in the third trimester (n = 155 low-risk women and N = 82 high-risk women in the second trimester). Results: The high-risk-for-PE women exhibited more depressive symptoms compared to the low-risk women in the second but not in the third trimester. No differences were observed between the two groups in other distress symptoms or in the women’s evaluation of their experience of pregnancy. The high-risk group reported greater health behaviour changes compared to the low-risk group, but this was moderated by depression levels. Conclusions: Overall, pregnant women reported positive attitudes towards first-trimester PE screening, despite transient depressive symptoms. This study offers supportive evidence concerning the appropriateness of PE screening in ethical terms.

## 1. Introduction

Pre-eclampsia (PE) is a multisystem disorder that affects 2–5% of pregnancies and is one of the main causes of maternal and foetal morbidity and mortality [1,2] causing 12% of all maternal deaths globally [3]. There have been numerous published approaches to screen for PE using a variety of biomarkers with varying degrees of efficacy [4,5,6]. However, given the complex nature of this multisystem disorder, the most effective screening method published for the first-trimester screening for PE uses a combination of maternal factors and biochemical and biophysical markers. The competing risk model from the ASPRE study uses gestational age at the time of delivery for PE as a continuous variable. Bayes’ theorem is then applied to combine prior information from maternal characteristics, obstetric and medical history with the MoM values of uterine artery pulsatility index (PI), mean arterial pressure (MAP), serum PAPP-A, and placental growth factor (P1GF). Screening by maternal factors with biochemical and biophysical markers yields detection rates of all PE and PE requiring delivery before 37 and 34 weeks’ gestation of 40%, 63%, and 76%, respectively, at a false-positive rate of 5% and 54%, 75%, and 88%, respectively, at a false-positive rate of 10% [7,8].

First-trimester screening for PE offers opportunities for the early identification of women at high risk, who can then be offered intensive surveillance and aspirin prophylaxis starting from the first trimester of pregnancy [9,10,11]. However, the integration of first-trimester PE screening into routine pregnancy care has not yet happened in the UK. Apart from practicality and costs, it is not known how feasible such an integration would be [12], and studies regarding women’s acceptance of introducing this PE screening into the first-trimester ultrasound scan are scarce [13,14]. Additionally, potential psychological harms and ethical concerns have also been raised. It has been suggested that being labelled as “high-risk” can alter the experience of pregnancy and introduce stress, worry, and anxiety, and in so-called “false positive” women, there would be additional antenatal visits and unnecessary medical prophylaxis [15].

To date, there have only been two studies [16,17] that have examined the psychological impact of being identified as “high risk” following the first-trimester PE screening. Simeone and colleagues [16] examined the changes in anxiety levels in women screened to be at high risk for PE and in low-risk women. No differences in the levels of maternal anxiety were identified neither immediately following the identification of the “high-risk” status nor subsequently, in the second or third trimester, between the two risk groups [16]. In a small qualitative study by Harris et al. [17], it was found that women broadly welcomed PE screening and the increase in pregnancy monitoring associated with the high-risk status. However, they also reported that, in those identified as “high risk”, the experience of pregnancy was altered from being a normal life event to becoming a source of worry. Many of the high-risk women in Harris and colleagues’ study expressed willingness to engage in efforts to reduce their risk for PE through health behaviour changes, which led the authors to suggest that there was potential to use the PE screening test as a basis for improving women’s health more broadly. A small sample of only 10 high-risk women employed in this qualitative study precluded full understanding of the impact of the “high-risk” status on women’s experience of pregnancy, their worry and, generally, the acceptability of the first-trimester PE screening.

There is currently a scarcity of research regarding the factors that impact women’s health behaviours before and during pregnancy [18,19]. Age, education, and pregnancy intentions (planned vs. unplanned pregnancies) were identified as relevant in a systematic review by Hillier and Olander [18]. Furthermore, a recent study [20] demonstrated that maternal coping, anxiety, and, in particular, maternal depression were significantly associated with health-promoting and health-harming behaviour in pregnant women. It is currently unknown what might be the impact of PE screening on pregnant women’s health behaviours [21].

The aim of the current study was to extend the understanding of the psychological impact of the first-trimester PE screening on women. Simeone and colleagues [16] examined the impact of the first-trimester PE screening on women’s anxiety levels solely. In our study, apart from anxiety, we evaluated the impact of the high-risk status on women’s depression levels, physical and mental health, and worry about their own and their baby’s health. Furthermore, we investigated whether high-risk PE status was associated with greater health behaviour changes in pregnancy; we hypothesised that women identified to be at high risk for PE would report more health behaviour changes compared to low-risk women, but this would be moderated by maternal depression, which would exert a negative impact on women’s reported health behaviour changes. Finally, we examined whether women’s experience of pregnancy and their attitudes towards PE screening differed between the low- and high-risk-for-PE groups.

## 2. Materials and Methods

### 2.1. Study Design and Sample

This study was nested within the ASPRE (Aspirin for Evidence-Based Pre-eclampsia Prevention) trial; the study inclusion criteria and procedure were published previously [10]. The ASPRE trial enrolled women from the participating centres in six different European countries. Out of 26,941 women with singleton pregnancies who underwent screening, 2971 (11%) were found to be at high risk of preterm PE; of these, 2641 met the eligibility criteria. However, 865 (32.8%) declined to participate in the trial and a further 152 (8.6%) women withdrew consent after randomisation. The women’s reasons for declining trial participation were described in an earlier study that included a UK-based sample [22].

The current study was conducted in the UK in two ASPRE-trial-participating hospitals. The only departure from the described ASPRE trial study inclusion criteria [6] was the necessity to comprehend written and spoken English. In the current study of the psychological evaluation, a longitudinal study design with three assessment points: before the first-trimester PE screening (time 1), at 22 weeks (time 2), and at 30 weeks of pregnancy (time 3), was employed. Participants were retained in the sample if they completed at least two out of three study questionnaires. 

Consecutive pregnant women (n = 585) attending their 11–14 weeks ultrasound appointment at a London hospital participating in the ASPRE trial were offered the opportunity to be screened for preterm-PE risk status and those with the high-risk status were invited to participate in the trial and in the psychological study. Out of 585 women, 225 (38.4%) completed the first study questionnaire; following the exclusion of women (n = 6) who did not meet the study inclusion criteria (e.g., twin pregnancies or pregnancy loss diagnosed during ultrasound examination), 197 (87.2%) were identified as low risk for preterm PE, and 28 (12.8%) were identified as high risk for preterm PE. 

Because of the few high-risk cases in the recruited sample, additional high-risk women were recruited from another ASPRE-trial-participating hospital in London (N = 54), who completed questionnaires in the second trimester. Out of the 197 questionnaires completed by the low-risk women before screening, 155 were returned in the second trimester; the total number of returned questionnaires by the high-risk group was 82 (Table 1). In the third trimester, completed questionnaires were received from 87 low-risk and 35 high-risk participants.

### 2.2. Procedure

Women attending their routine 11–13 ultrasound appointment were offered the opportunity to be screened for preterm-PE risk status as part of the ASPRE trial. They were informed about the aims of the psychological evaluation that was carried out independently from the medical research. Participants who consented to taking part were asked to complete study questionnaires on three occasions during their course of pregnancy. The first questionnaire (time 1) was completed at 11–13 weeks, before PE screening. The results of the PE screening test were given to the participants during the first-trimester ultrasound examination. The result was either high risk for PE (>1 in 100) or low risk for PE. The high-risk group received medical counselling from a doctor regarding their risk status, its meaning, and the consequences. As part of the ASPRE trial, women at high risk for preterm PE were offered to be randomised into one of two groups: (a) daily low-dose aspirin (150 mg) until 36 weeks’ gestation or (b) a placebo group. A second questionnaire (time 2) was completed at 20–24 weeks when the women attended a hospital appointment or were mailed the questionnaires along with a prepaid envelope. The third questionnaire (time 3) at 28–32 weeks was posted to the participants’ home address. The local National Health Service research ethics committee granted ethics approval for this study (ref: 14/LO/1238).

### 2.3. Materials

*Demographic- and pregnancy-related information* (age, ethnicity, educational attainment, employment status, marital status, history of previous emotional difficulties, primiparous, and natural/assisted conception pregnancy) was collected via a study-designed questionnaire.

*Anxiety* was assessed using the six-item version (STAI-S) [23] of the State–Trait Anxiety Index [24] (STAI). STAI-S6 scores range from 20 to 80, with higher scores suggesting higher levels of anxiety. Excellent psychometric properties have been reported for STAI-S6 [25]. In our sample, Cronbach’s alpha ranged from 0.83 to 0.89 across the three time points.

*Depressive symptoms* were measured using the eight items of the Edinburgh Postnatal Depression Scale (EPDS), a highly valid and reliable screening tool for perinatal depression [26]. In the current study, the ten-item scale was reduced to eight items (omitting two items, “I have been so unhappy that I have been crying” and “The thought of harming myself has occurred to me” as they were recommended to be removed by the ethics board). The total scores available ranged from 0 to 24 for the eight items of the EPDS, with higher scores indicating more depressive symptoms. Cronbach’s alpha ranged from 0.85 to 0.92 across the three time points.

*Worry* was assessed using the modified version of the Cambridge Worry Scale (CWS) [27]; four worry items deemed relevant to the current study were utilised (“worry about own health/giving birth/having labour too early/baby’s health”) with a five-point Likert scoring for each item (0—not a worry to 5—major worry). A principal factor analysis conducted on the four items revealed that they loaded on one factor (eigenvalue > 1) explaining 62% of the variance. The summary score for the four items ranged from 0 to 20, with higher scores indicating greater worry. Cronbach’s alpha ranged from 0.77 to 0.80 across the three time points, indicating good internal reliability.

*Physical and mental health status* was assessed using the Short Form-12 (SF12) Health Survey Questionnaire [28], a short, generic measure of subjective health status. (Changes were made to SF-12 in terms of adding an extra response choice of “ A good bit of the time”, and changing the wording of items 2a, 4, and 6 which were not relevant to pregnant women; this might have affected the external validity of the survey. These modifications were not made with the consent or approval of OptumInsight Life Sciences (QualityMetric.) The SF-12 includes 12 items assessing mental (MCS-12) and physical health (PCS-12), with higher scores indicating a better health status. Across the three time points, Cronbach’s alpha ranged from 0.67 to 0.82 for physical health and from 0.72 to 0.84 for mental health.

*The impact of PE screening status on health behaviours* was adapted from Orbell et al. [29]. Five items assessed self-reported changes in diet and exercise: “I have been eating more healthily/taking more fibre/less salt/less fat/more exercise than I used to before PE screening”. Each statement was followed by a 1–6 Likert-style scoring system (do not agree to agree very strongly, with the option of no change from before PE screening), with higher scores representing a greater degree of change. A principal factor analysis conducted on the five items revealed that they loaded on one factor (eigenvalue > 1), explaining 70.5% of variance. The five questions were administered in the second and third trimester to both the low-risk and high-risk groups. Strong internal reliability was evident: Cronbach’s alphas of 0.89 at time 2 and 0.91 at time 3 were obtained.

*The overall experience of pregnancy and attitude to screening*: Four study-designed items examining the overall experience of pregnancy and women’s attitude to screening were included in the third trimester for both low- and high-PE-risk groups. The women were asked to state how their experience of pregnancy (very/moderately negative, unsure, or moderately/very positive) was; whether, in their view, the benefits of screening for PE in pregnancy outweighed the costs (yes definitely, moderately, little, no, or unsure); whether they would request a PE screening in a future pregnancy (if it were not offered) (yes/no/unsure); and whether they would recommend PE screening to other pregnant women (yes/no/unsure).

### 2.4. Data Analysis

To examine the impact of first-trimester PE screening and prevention throughout pregnancy, we first examined the background differences across relevant demographic and obstetrics factors between the low- and high-risk-for-PE women (at time 2) using Student’s *t*-test and Pearson’s chi square test. We then performed univariate comparisons between women at high risk and low risk for PE on physical and mental health, anxiety, depressive symptoms, worry, lifestyle behaviours (i.e., changes in diet or exercise), and the overall attitudes and acceptability of PE screening and prevention. A multiple linear regression analysis was performed to test the association between PE risk status (high vs. low risk) and the above-mentioned variables for each assessment time (time 1, time 2, and time 3), accounting for the confounding effects of maternal education, employment, relationship status, whether it was their first child, and previous emotional difficulties; these were identified as potential confounders due to the significant differences between the high- and low-risk groups across these variables.

Given the identified differences between the low- and high-risk-for-preterm-PE groups regarding health behaviour changes, two-way analyses were carried out to examine the interaction at time 3 between risk status (high vs. low PE risk) and depression on lifestyle changes. Continuous variables were standardized before being inserted and multiplied in the regression model and the following variables were inserted as covariates in order to statistically control for their confounding effects: maternal education, employment, relationship status, whether it was their first child, and previous emotional difficulties.

Finally, to ensure results were not inflated by the two-step recruitment of participants in the high-risk group, we performed the same analytic strategy as described above on the subsample of high-risk and low-risk participants recruited and longitudinally assessed across *time 1, time 2, and time 3* (thus excluding the second high-risk sample). In this subsample, a “true” prospective, longitudinal design was present. Their demographic characteristics are presented in the Supplementary Materials, Table S1.

All analyses were performed using R [30]. Two-tailed *p* values were used and *p* < 0.05 was considered statistically significant.

## 3. Results

The descriptive statistics and group differences in physical and mental health indexes, anxiety, depression, and worry between the high-risk- and low-risk-for-PE groups across pregnancy (first, second, and third trimester) are shown in Table 2. When accounting for confounding variables, compared to the low-risk-for-PE group, high-risk pregnant women reported more symptoms of depression in the second trimester (b = 1.51, SE = 0.63, *t*(220) = 2.40, and *p* = 0.017), and better physical health in the first (b = 3.43, SE = 1.34, *t*(209) = 2.56, and *p* = 0.011) and third trimester (b = 5.07, SE = 2.41, *t*(106) = 2.10, and *p* = 0.038). There were no other significant differences between the two groups. Similar results were observed in the “true” longitudinal subsample where differences between high- and low-risk women nearly reached significance in the second trimester (adjusted *p* value = 0.051), with high-risk women reporting more depressive symptoms in the second but not in the first or the third trimester (Supplementary Materials Table S2).

We examined whether there were any differences across the psychological outcomes between the women who completed the second trimester questionnaire as well as the third trimester questionnaire and those who did not complete the third questionnaire (for both high- and low-risk groups separately). A similar pattern was observed in that in both low- and high-risk groups, those who did not return the third questionnaire were significantly more depressed in the second trimester (*p* < 0.05 in both groups). Low-risk women who did not return the third questionnaire were also more likely to be anxious (*p* < 0.05) in the second trimester (*p* < 0.05). No other differences were identified.

Table 3 shows the group differences in health behaviours (i.e., changes in diet and exercise) between the high-risk- and low-risk-for-PE groups in the second and third trimesters. The high-risk-for-PE group reported greater lifestyle-related changes compared to the low-risk-for-PE group in the second (b = 3.31, SE = 1.11, *t*(219) = 2.99, and *p* = 0.003) and third trimester (b = 4.08, SE = 1.67, *t*(103) = 2.45, and *p* = 0.016). Similar results were observed in the longitudinal subsample where differences between the high- and the low-risk women were significant in the second trimester but failed to reach significance in the third trimester (Supplementary Materials, Tables S3 and S4).

**Figure 1:**
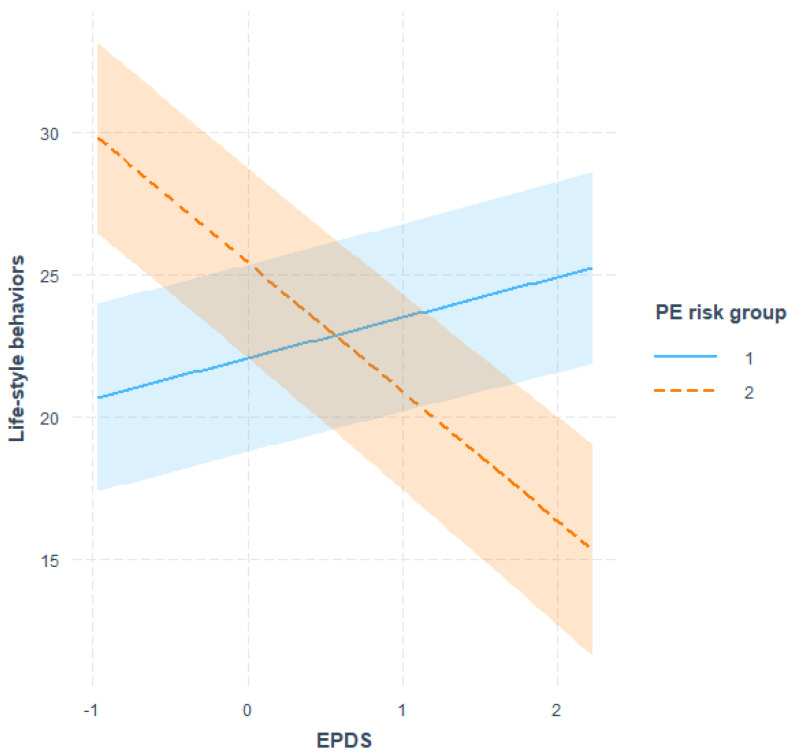
Simple slope analysis for Risk group x Depression interaction at time 3 on Life-Style Behaviours. Note: PE = preeclampsia; PE risk group 1 = low risk of preeclampsia/PE risk group 2 = high risk of preeclampsia.

We then tested our hypothesis that depression levels would moderate the impact of PE health status on health behaviour changes, with women scoring high in depressive symptoms being less likely to report changes in health behaviours subsequent to their high-risk-for-PE screening test result. The findings confirmed our hypothesized relationship and a significant two-way interaction between PE screening risk (high vs. low) and maternal depression on health behaviours (interaction term: beta = −0.33, *p* = 0.002) was identified. Indeed, under the condition of low PE risk, no significant differences in lifestyle changes were observed between women with high or low depressive scores; on the contrary, in the high-PE-risk group, women reporting low depression scores endorsed significantly more health behaviour changes compared to women with higher depression scores. Figure 1 depicts a graphic representation of this effect. This was also observed in the longitudinal subsample (Supplementary Materials Figure S1).

Table 4 shows the group differences in pregnant women’s experience of pregnancy and the attitudes towards screening for PE in the third trimester. All high-risk-for-PE pregnant women, except one, reported that their experience of pregnancy was positive, and this was not statistically different from the experience of low-risk pregnant women. A nonsignificant trend was identified towards low-risk pregnant women expressing more perceived benefits of screening compared to high-risk-for-PE pregnant women. No significant differences were identified between the two groups in terms of future requests for screening and recommendation of PE screening; in both groups, the majority expressed that they would request PE screening in a future pregnancy and that they would recommend PE screening to others.

## 4. Discussion

Although it has been well documented that first-trimester PE screening is of clinical value [8,9,10,11], studies examining its psychological impact are scarce. The only quantitative study to date by Simeone and colleagues [16] demonstrated no adverse impact of the “high-risk” PE status on women’s anxiety during pregnancy. Our study first quantitatively examined the impact of the high-risk-for-PE status across a range of psychological outcomes including anxiety, depression, physical and mental health, and worry about own and baby’s health, as well as women’s health behaviours, pregnancy experience, and attitudes towards PE screening.

The study findings revealed that, among the various psychological distress outcomes considered, women identified to be at high risk for PE during the first-trimester screening exhibited more depressive symptoms at 22 weeks of pregnancy compared to low-risk women. These symptoms were transient as no significant differences between the low-risk- and high-risk-for-PE groups were identified in the third trimester. No differences between the two groups in anxiety, mental health, or worry about own or baby’s health were identified at any point of assessment. There were differences in the physical health in the first and third trimesters between the two groups of women, but these could be seen as cohort effects rather than “true” differences as such differences were present at baseline, before the PE screening test result was known. Our findings showcasing no elevations in anxiety levels are aligned with the findings of Simeone and colleagues, who reported that there is no adverse impact of the “high-risk” PE status on women’s anxiety levels [16]. Transient increases in anxiety and worry following a high-risk screening test result regarding foetal health (e.g., Down’s syndrome) but not maternal health conditions (e.g., gestational diabetes) have been reported in the literature (for a review, see Harris et al. [21]). High-risk screening health status inevitably challenges the expectancy of a “normal”, healthy pregnancy and can be subjectively experienced as a threat and/or a loss experience that gives rise to anxiety or depressive symptoms. It has been suggested that a high-risk status for conditions where there is a sense of perceived control over the outcome of that threat (e.g., maternal health conditions) will be experienced as less distressing than when the threat is seen as less “controllable” (i.e., foetal health conditions) [17,21].

Our study identified that the high-risk-for-PE group reported to have made more changes to their health behaviours compared to women at low risk for PE. The questions relating to health behaviours mostly focused on dietary changes (eating more healthily; consuming less salt and fat and more fibre). It is important to note that medical professionals did not explicitly recommend changes to health behaviours to women at high risk for PE during antenatal screening. In line with suggestions that pregnancy can offer “teachable moments” for health behaviour change [17,21], it is possible that the positive PE screening result led high-risk women to re-evaluate their health behaviours and initiate change as a means of coping, aimed at “gaining control” over the threatening situation. The perception of control is seen as one of the central constructs in the psychological models of people’s representations and coping with health threats (e.g., the common sense model [31,32]). Although Harris and colleagues [21] suggested that positive behaviour changes were not universally present in their sample of ten high-risk women, our results revealed that such positive health behaviour changes were significantly greater amongst high-risk women compared to those at low risk, both in the second and the third trimester.

We hypothesized that engagement in health behaviour change would be moderated by women’s depression levels. This hypothesis was supported as, amongst high-risk women, those reporting higher depression scores were significantly less likely to report having made changes to health behaviours in the second and third trimester in comparison to women with lower depression scores. Thus, although a high-risk test result may trigger changes in health behaviours, this could be hindered by the depressive symptoms experienced by the woman. This is an important finding as it suggests that depressive states may reduce the sense of agency and behavioural control that women will display during pregnancy, and that affected women will be less likely to engage in changes in health behaviours that would support their own health and that of their unborn child. This finding is concordant with a recent study [20] on pregnant women where depression was found to be the strongest independent predictor (more so than anxiety or coping strategies) of health-harming nutritional choices.

We found that the majority of women, regardless of their PE risk status, reported having had a positive experience of pregnancy and most of them endorsed the benefits of screening as outweighing the perceived costs. The majority also stated they were likely to request PE screening in a future pregnancy and recommend it to pregnant friends and relatives. Others have also reported that, generally, pregnant women, as well as those at high risk for PE, hold positive views in relation to PE screening in pregnancy [13,21] and this is concordant with other studies on pregnant women that have reported positive attitudes to screening for both foetal and maternal conditions [33,34]. It is also important to note that a minority of women (20% in the high-risk group vs. 5% in the low-risk group) reported that they did not think that the benefits of screening outweighed the costs. It was not possible from our study to determine why a minority of women held such beliefs. Future studies employing qualitative methodology would be able to elucidate further the reservations regarding the PE screening that some women may hold.

Our study findings offer unique insights not only towards a better understanding of the psychological impact of first-trimester PE screening on pregnant women, but also offer supportive evidence concerning the appropriateness of such a screening in ethical terms. From an ethical standpoint, the benefits of introducing novel screening programmes should be weighed against the potential harm for those women falsely screened as positive [35]. In our study, among the many psychological outcomes considered, we only found a transient negative impact on maternal depressive symptoms among the high-risk-for-PE women. The implications of this, and the finding of fewer health behaviour changes being reported among women with higher depression scores, are in terms of the provision of antenatal support. Screening for depression and supporting women who exhibit elevated depression scores are important to ensure better maternal health and well-being both pre- and postnatally [36]. Such improvements are likely to benefit mothers in terms of changes to their health behaviours, self-care, and well-being, which in turn would optimise the outcomes for their unborn child.

Our findings need to be considered in light of the study limitations. Despite the considerable sample size overall, attrition in the study was significant and led to small sample sizes, particularly in the high-risk group during the last evaluation phase. The low incidence of the high-risk-for-PE status and the adopted longitudinal study design offered further challenges to the recruitment of the high-risk-for-PE cohort. Due to the loss of participants over time and the variability in participants at each time point of assessment, we were unable to treat our data as “truly” longitudinal and our data analytic strategy was adjusted to address that shortcoming in the main data set. We then also analysed the smaller subsample of the high-risk women who met the criteria of the prospective longitudinal design and were able to identify a very similar set of findings. Issues relating to recruitment and attrition will remain a challenge for researchers examining the impact of PE screening. The strength of our study is that a large number of confounders were included in the analyses, which gives confidence to the validity of our findings. Our study sample was predominantly White and well educated, and it is not possible to know to what extent our findings can be generalised to more ethnically, socially, and economically diverse populations. In addition, our study was nested within the ASPRE trial, an RCT for the prevention of PE using low-dose aspirin vs. a placebo. The women in our study were “blind” to whether they were taking aspirin or a placebo, but all of the high-risk women were offered additional monitoring and scans throughout pregnancy, which they found reassuring and beneficial [18]. It is not known to what extent such extra care might have contributed to offsetting the potential negative psychological impact of the high-risk-for-PE status. Further studies are needed to examine whether our findings can be verified in routine clinical practice.

## 5. Conclusions

Our study offers novel insights concerning the psychological impact of the first-trimester screening for PE. The findings revealed that, overall, pregnant women had positive attitudes towards PE screening, and that the pregnancy experiences of those identified to be at high risk following the first-trimester PE screening were comparable to those identified as low risk. High-risk women reported more positive health behaviour changes compared to low-risk women subsequent to the positive test result, but this was moderated by the depression levels. Although no adverse effects of the “high-risk status” for anxiety, physical and mental health, and worry were identified, there was a transient increase in depressive symptoms in the second trimester of pregnancy among the high-risk women. Future studies conducted in routine clinical practice and utilising larger and more diverse samples would provide a definitive answer as to whether this increase in depressive symptoms is indeed of a transient nature or not.

## Figures and Tables

**Table 1:** Baseline sociodemographic characteristics of the included population (N = 282) by group.

	Low-Risk PE (*n* = 197)	High-Risk PE (*n* = 82)	*p* Values
Maternal age (years)	33.09 (4.35)	31.84 (5.44)	0.06
Maternal ethnicity			
White Black/South Asian/other	138 (70) 59 (30)	61 (72) 24 (28)	ns
Maternal education			
O levels A levels Graduate degree Pg degree	14 (7) 36 (18) 93 (47) 53 (27)	16 (19) 13 (15) 35 (43) 20 (24)	0.044
Maternal employment			
employed unemployed/homemaker/student/other	170 (86) 27 (14)	69 (81) 26 (20)	0.010
Relationship status			
married or living with partner single, separated, divorced, other	190 (96) 7 (4)	71 (84) 14 (16)	<0.001
Previous emotional difficulties (no)	143 (73)	68 (80)	ns
First child (no)	98 (50)	18 (21)	<0.001
Conception (natural)	189 (96)	79 (92)	ns

Note. data are given as No. (%), mean (SD); *p*-values were calculated using Student’s *t*-test and Pearson’s chi square test; PE = pre-eclampsia. ns = not significant.

**Table 2:** Group (low and high PE risk) differences in mental health indexes across pregnancy time points.

	**Time 1**				
	**Low-Risk PE** **(*n* = 197)**	**High-Risk PE** **(*n* = 28)**	***p* Values**	**Adjusted** ***p* Values**	**Cohen’s *d*** **[95% CI]**
SF—physical health	50.33 (6.59)	53.04 (4.27)	0.040	0.011	0.43 [0.029–0.824]
SF—mental health	51.81 (10.04)	51.68 (7.01)	0.947	0.926	0.013 [−0.409–0.38]
Anxiety	32.34 (11.18)	31.55 (10.32)	0.711	0.469	−0.71 [−0.143–0.65]
Depression	4.32 (3.83)	4.13 (4.41)	0.914	0.635	−0.05 [−0.445–0.347]
Worry	8.71 (4.61)	8.32 (4.56)	0.676	0.429	−0.09 [−0.481–0.311]
	**Time 2**				
	**Low-risk PE** **(*n* = 155)**	**High-risk PE** **(*n* = 82)**	***p* values**	**Adjusted** ***p* values**	**Cohen’s *d*** **[95% CI]**
SF—physical health	47.07 (7.28)	46.13 (8.87)	0.406	0.367	0.12 [−0.387–0.148]
SF—mental health	49.75 (8.49)	47.17 (11.36)	0.052	0.061	−0.27 [−0.538–0.001]
Anxiety	33.12 (11.78)	36.09 (13.40)	0.094	0.205	0.24 [−0.028–0.509]
Depression	4.88 (4.033)	6.83 (5.65)	0.007	0.017	0.42 [0.149–0.689]
Worry	8.57 (4.07)	8.92 (5.28)	0.596	0.829	0.08 [−0.19–0.345]
	**Time 3**				
	**Low risk PE** **(*n* = 87)**	**High risk PE** **(*n* = 35)**	***p* values**	**Adjusted** ***p* values**	**Cohen’s *d*** **[95% CI]**
SF—physical health	42.89 (10.49)	46.92 (8.96)	0.055	0.038	0.40 [0.004–0.795]
SF—mental health	48.99 (10.16)	49.30 (9.48)	0.88	0.908	−0.205 [−0.599–0.188]
Anxiety	34.75 (13.96)	29.90 (10.43)	0.041	0.332	−0.37 [−0.767–0.023]
Depression	5.57 (5.94)	5.11 (4.90)	0.665	0.823	−0.08 [−0.474–0.311]
Worry	7.51 (4.47)	7.03 (4.25)	0.584	0.399	−0.11 [−0.501–0.284]

Note. Data are given as mean (SD); *p*-values were calculated using Student’s *t*-test; *p* values adjusted for: maternal age, education, employment, parity, relationship status, and previous emotional difficulties; PE = pre-eclampsia; SF = short form.

**Table 3:** Group (low and high PE risk) differences in lifestyle-related behavioural changes after PE screening at times 2 and 3.

**Time 2**					
	**Low-Risk PE** **(*n* = 155)**	**High-Risk PE** **(*n* = 82)**	***p* Values**	**Adjusted** ***p* Values**	**Cohen’s *d*** **[95% CI]**
Lifestyle behaviours	7.71 (7.45)	11.94 (7.56)	<0.001	0.003	0.57 [0.292–0.837]
**Time 3**					
	**Low risk PE** **(*n* = 84)**	**High risk PE** **(*n* = 34)**	***p* values**	**Adjusted** ***p* values**	**Cohen’s *d*** **[95% CI]**
Lifestyle behaviours	5.96 (6.44)	10.47 (8.34)	0.006	0.016	0.64 [0.241–1.042]

Note. Data are given as mean (SD); *p*-values were calculated using Student’s *t*-test *p* values adjusted for: maternal age, education, employment, parity, relationship status, and previous emotional difficulties.

**Table 4:** Group (low and high PE risk) differences in attitude toward PE screening at time 3.

	Low Risk PE (*n* = 83)			High Risk PE (*n* = 35)			*p* Values
Attitude to Screening	Unsure	Negative	Positive	Unsure	Negative	Positive	
Experience of pregnancy	1 (1)	8 (9)	75 (88)	0 (0)	1 (3)	33 (94)	0.58
Benefits of screening	12 (14)	4 (5)	68 (82)	3 (9)	7 (20)	24 (69)	0.06
Future request for screening	24 (29)	6 (7)	54 (65)	6 (17)	5 (14)	23 (66)	0.26
Recommend screening	16 (19)	2 (2)	66 (80)	8 (23)	2 (6)	24 (69)	0.52

Note. Data are given as N (%); *p*-values are calculated using chi square test.

## Data Availability

Data are available on request to the corresponding author.

## Supplementary Materials

The following supporting information can be downloaded at: https://www.mdpi.com/article/10.3390/ijerph20075418/s1. Table S1. Group differences in baseline socio-demographic characteristics (N = 225); Table S2. Group differences in mental-health indexes across the three assessment points; Table S3. Group differences in well-being and self-care indexes across pregnancy’s timepoints; Table S4. Simple effects of Risk group: parameter estimates; Figure S1. Simple slope analysis for Risk group × Depression interaction at time 3 on Life-Style Behaviours.
