# Supplementary material for: Psychological Impact and Women’s Evaluation of the First-Trimester Pre-Eclampsia Screening and Prevention: ASPRE Trial
Source: Int J Environ Res Public Health. 2023 Apr 6;20(7):5418. doi: 10.3390/ijerph20075418 (PMC10094560; doi:10.3390/ijerph20075418)
Supplement: Supplementary file 1 [file ijerph-20-05418-s001.zip › ijerph-2149066-supplementary.pdf]

International Journal of
Environmental Research and Public Health

MDPI

Supplementary Materials:

Sample of N = 225 first-trimester pregnant women prospectively and longitudinally assesses across the three assessment points.

Table S1. Group differences in baseline socio-demographic characteristics (N=225)

	**Low-risk (*n* = 197)**	**High risk (*n* = 28)**	***P* values**
Maternal age (years)	33.09 (4.35)	32.46 (4.57)	ns
Maternal education			
O levels	-	1	
A levels	14	2	ns
Graduate degree	36	6	
Pg degree	93	11	
	53	7	
Maternal employment			
employed	170	25	
unemployed/homemaker/	3	-	ns
student/other	24	3	
Relationship status			
married or living with partner	190	23	**0.007**
single, separated, divorced, other	7	5	
Emotional difficulties (no)	143	24	ns
Parity (no)	98	7	**0.034**
Conception (natural)	189	25	ns

Note. Data are given as No. (%), mean (SD)
P-values are calculated using Student’s t-test, Pearson’s chi-square test
PE = preeclampsia
ns = not significant

Table S2. Group differences in mental-health indexes across the three assessment points.

	1st trimester			
	Low risk (n = 197)	High risk (n=28)	*P* values	Adjusted *P* values
SF- physical health	74.36 (1.99)	75.13 (1.39)	**0.015**	0.064
SF- mental health	82.64 (3.18)	83.45 (3.29)	0.228	0.337
Anxiety	32.34 (11.18)	31.55 (10.32)	0.711	0.469
Depression	4.32 (3.83)	4.13 (4.41)	0.914	0.635
Worry	8.71 (4.61)	8.32 (4.56)	0.676	0.429

Note. Data are given as mean (SD); P-values are calculated using Student's t-test
P values adjusted for: maternal age, education, employment, parity, relationship status and previous emotional difficulties

	2nd trimester			
	Low risk (n = 155)	High risk (n= 25)	*P* values	Adjusted *P* values
SF- physical health	72.82 (2.56)	73.35 (2.19)	0.289	0.421
SF- mental health	82.89 (3.36)	83.11 (3.56)	0.784	0.713
Anxiety	33.12 (11.78)	33.61 (13.15)	0.864	0.402
Depression	4.88 (4.03)	6.00 (6.17)	0.389	0.051
Worry	8.57 (4.06)	6.96 (4.00)	0.072	0.036

Note. Data are given as mean (SD); P-values are calculated using Student's t-test
P values adjusted for: maternal age, education, employment, parity, relationship status and previous emotional difficulties

	3rd trimester			
	Low risk (n = 87)	High risk (n = 20)	*P* values	Adjusted *P* values
SF- physical health	72.24 (3.09)	73.57 (2.19)	**0.037**	0.059
SF- mental health	82.71 (3.95)	83.17 (3.61)	0.642	0.560
Anxiety	34.75 (13.96)	29.74 (10.02)	0.062	0.967
Depression	5.57 (5.94)	5.05 (5.47)	0.709	0.582
Worry	7.51 (4.47)	6.65 (3.6)	0.369	0.037

Note. Data are given as mean (SD); P-values are calculated using Student's t-test
P values adjusted for: maternal age, education, employment, parity, relationship status and previous emotional difficulties

Table S3. Group differences in well-being and self-care indexes across pregnancy's timepoints

	**2nd trimester**			
	Low risk (n = 155)	High risk (n= 25)	*P* values	Adjusted *P* values
Life-style changes	7.71 (7.45)	12.13 (6.74)	< 0.001	0.023

Note. Data are given as mean (SD); P-values are calculated using Student's t-test
P values adjusted for: maternal age, education, employment, parity, relationship status and previous emotional difficulties

	**3rd trimester**			
	Low risk (n = 87)	High risk (n= 20)	*P* values	Adjusted *P* values
Life-style changes	5.97 (6.44)	10.05 (8.44)	0.059	0.143

Note. Data are given as mean (SD); P-values are calculated using Student's t-test
P values adjusted for: maternal age, education, employment, parity, relationship status and previous emotional difficulties

Supplement Moderation analysis

Figure S1. Simple slope analysis for Risk group x Depression interaction at time 3 on Life-Style Behaviours.

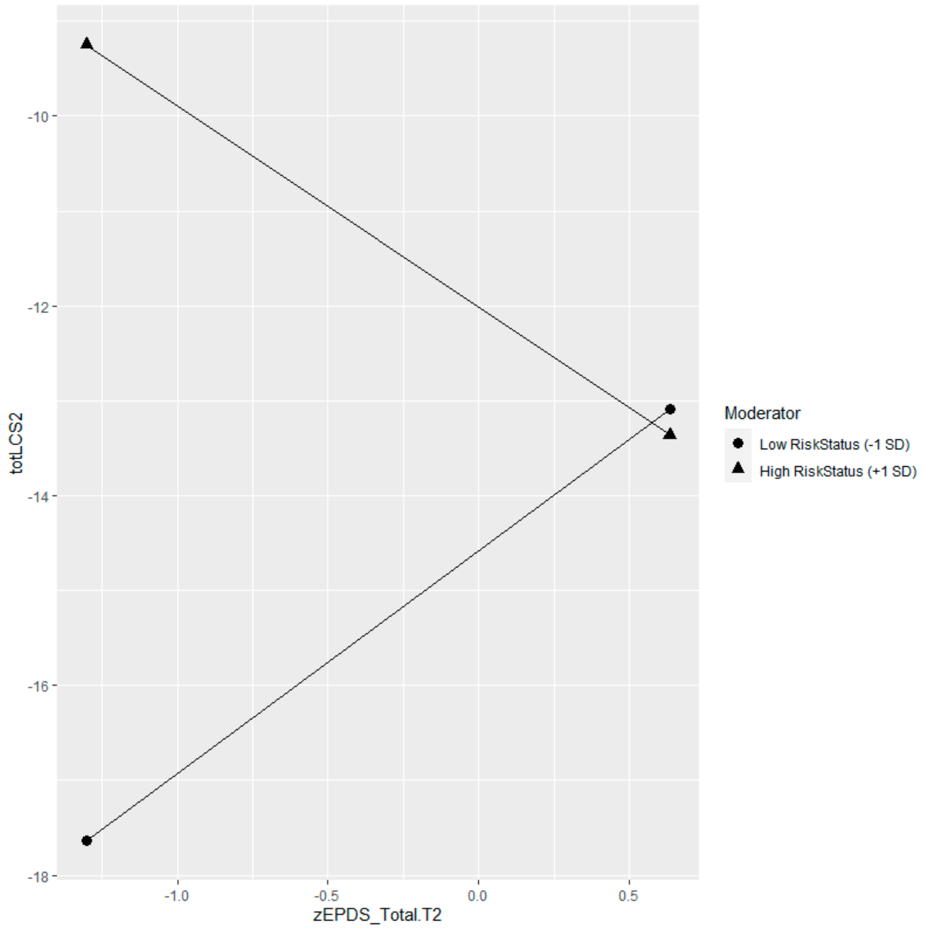

Table S4. Simple effects of Risk group: parameter estimates.

	**Estimate**	**SE**	**t**	***p***
Low Risk	1.982436	2.007804	0.9873656	0.329
High Risk	-2.503026	1.420016 -	-1.7626743	0.085
